# A fragment of cell adhesion molecule L1 reduces amyloid-β plaques in a mouse model of Alzheimer’s disease

**DOI:** 10.1038/s41419-021-04348-6

**Published:** 2022-01-10

**Authors:** Junkai Hu, Stanley Li Lin, Melitta Schachner

**Affiliations:** 1 Center for Neuroscience, Shantou University Medical College, 22 Xin Ling Road, Shantou, Guangdong 515041 China; 2 Deaprtment of Cell Biology, Shantou University Medical College, 22 Xin Ling Road, Shantou, Guangdong 515041 China; 3 Guangdong Provincial Key Laboratory for Breast Cancer Diagnosis and Treatment, Shantou University Medical College, Shantou, China; 4 Keck Center for Collaborative Neuroscience, Department of Cell Biology and Neuroscience, School of Arts and Sciences, Rutgers, The State University of New Jersey, Piscataway, NJ 08854 USA

**Keywords:** Cell death, Cognitive ageing

## Abstract

Deposition of amyloid-β (Aβ) in the brain is one of the important histopathological features of Alzheimer’s disease (AD). Previously, we reported a correlation between cell adhesion molecule L1 (L1) expression and the occurrence of AD, but its relationship was unclear. Here, we report that the expression of L1 and a 70 kDa cleavage product of L1 (L1-70) was reduced in the hippocampus of AD (APPswe) mice. Interestingly, upregulation of L1-70 expression in the hippocampus of 18-month-old APPswe mice, by parabiosis involving the joining of the circulatory system of an 18-month-old APPswe mouse with a 2-month-old wild-type C57BL/6 mouse, reduced amyloid plaque deposition. Furthermore, the reduction was accompanied by the appearance of a high number of activated microglia. Mechanistically, we observed that L1-70 could combine with topoisomerase 1 (Top1) to form a complex, L1-70/Top1, that was able to regulate expression of macrophage migration inhibitory factor (MIF), resulting in the activation of microglia and reduction of Aβ plaques. Also, transforming growth factor β1 (TGFβ-1) transferred from the blood of young wild-type C57BL/6 mice to the aged AD mice, was identified as a circulating factor that induces full-length L1 and L1-70 expression. All together, these findings suggest that L1-70 contributes to the clearance of Aβ in AD, thereby adding a novel perspective in understanding AD pathogenesis.

## Introduction

Alzheimer’s disease (AD) is an irreversible neurodegenerative disease and is the most common cause of dementia among older adults [1, 2]. Clinically, the principal manifestations of AD patients are the progressive decline of memory and understanding, language and mental disorders, and the progressive weakening of cognition, including spatial and temporal judgment [3]. Whole-brain atrophy occurs, which is characterized by smaller volume, lighter weight, and thinner cortex, all of which are accompanied by amyloid β-protein (Aβ) deposits throughout the brain neuropil. Other histopathological features of AD include nerve fiber tangles caused by hyperphosphorylation of tau protein within neurons, diffuse inflammatory necrotic foci, and loss of neurons [4]. The amyloid cascade and tau hyperphosphorylation hypotheses were formed in the 1980s based on these AD pathological characteristics [5–8]. AD has been increasingly recognized to be an individual and social problem worldwide. Hence, the development of an AD treatment is urgently needed [9, 10]. We considered the function beneficial cell adhesion molecule L1 (L1) as a candidate that might alleviate some symptoms of AD. L1 is a transmembrane glycoprotein that participates in the mutual recognition and interconnection between nerve cells [11]. L1 also functions in neuronal survival and migration, neurite outgrowth, axonal fasciculation and targeting, synapse formation, synaptic plasticity, and myelination [12–18]. Previous studies reported on an association of L1 with AD: increased L1 levels were found in the cerebrospinal fluid of AD patients [19]. Also, L1 binds to Aβ and reduces Alzheimer’s disease pathology in mice when full-length L1-encoding adeno-associated virus is injected into the hippocampus and occipital cortex of AD mice expressing a mutant *APP* gene [20]. In frontal lobe tissue, L1 ameliorates some aspects of Aβ_1-42_ pathology in parallel with reducing protein kinase D1 (PKD1) function [21]. Through inhibiting Aβ_1-42_ formation and histone deacetylase 2 (HDAC2) expression, leading to downregulation of glucocorticoid receptor 1 activity, L1 can alleviate AD pathology [22]. L1 can also participate in the functional activity of the nervous system in different pathophysiological states [23, 24]. Furthermore, an L1 fragment with an apparent molecular weight of 70 kDa (L1-70), produced by cleavage by a serine protease, contains L1’s complete intracellular and part of the extracellular domain, and can enter mitochondria and the nucleus, contributing to changes in mitochondrial function and transcription [25–28]. Therefore, we hypothesized that L1 and, in particular, L1-70 could also attenuate AD pathogenesis.

We here report that hippocampal L1-70 expression in AD mice decreases Aβ deposition by combining with topoisomerase 1 (Top1) to form a complex that enters the nucleus to regulate the expression of macrophage migration inhibitory factor (MIF), resulting in activation of microglia for clearance of amyloid plaques. The fact that L1-70 reduces levels of Aβ clearance not only promotes our understanding of Aβ metabolism but also provides a novel perspective on the study of AD pathogenesis.

## Results

### Levels of L1-70 and full-length L1 are decreased in the hippocampus of aged APPswe mice showing increased deposition of Aβ

**Fig. 1:**
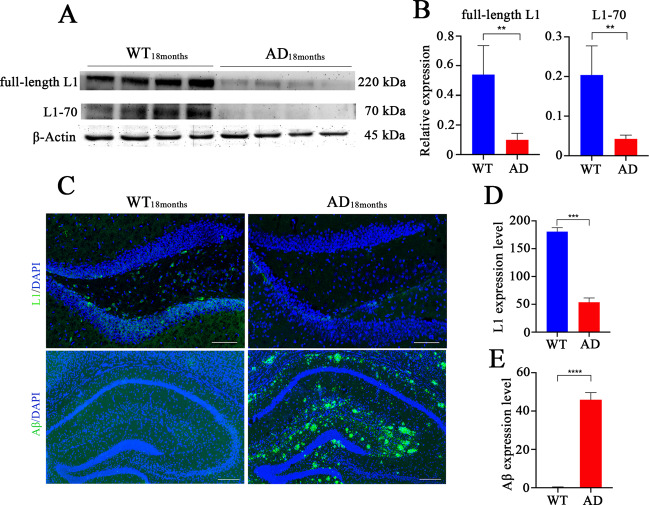
Expression of full-length L1, L1-70, and Aβ in the hippocampus of 18-month-old wild-type and APPswe mice. **A** Western blot analysis of hippocampal tissue homogenates of 18-month-old wild-type mice and APPswe mice. **B** Relative full-length L1 and L1-70 levels were calculated and normalized to β-Actin. Data were shown as means ± SD, **p < 0.01, Student’s *t*-test, *n* = 4 mice per group. **C** Representative immunofluorescence images of hippocampal tissue sections from 18-month-old wild-type and 18-month-old APPswe mice. Antibodies against L1 cytoplasmic domain and Aβ were used for immunostaining. Expression of L1 and Aβ in 18-month-old wild-type mice and 18-month-old APPswe mice are shown. **D**, **E** Full-length L1 and Aβ levels were calculated using ImageJ. Data were shown as means ± SD, ***p < 0.001, ****p < 0.0001, Student’s *t*-test, *n* = 4 mice per group. Scale bars: 100 µm.

L1 is increased in the cerebrospinal fluid of AD patients [19], attenuates the deposition of Aβ in the hippocampus of aged APPswe mice [20], and is cleaved into a 70 kDa fragment (L1-70) (Supplemental Fig. 1), that enters mitochondria and the nucleus [25, 26]. To gain an understanding between L1-70, L1, and Aβ deposition, we analyzed 18-month-old APPswe mice and 18-month-old wild-type mice. Western blots of hippocampus homogenates were probed with anti-L1 cytoplasmic domain, which revealed extensive expression of both L1-70 and L1 in the hippocampus of wild-type but not APPswe mice (Fig. 1A, B). In order to further understand the in situ expression of L1-70 and L1, and its relationship with Aβ deposition in the hippocampus, brains from 18-month-old wild-type and 18-month-old APPswe were fixed with formalin for paraffin sections, and immunofluorescence staining was carried out with anti-L1 cytoplasmic domain. The results showed that positive staining in the hippocampal neurons from aged wild-type mice and aged APPswe mice (Fig. 1C). However, the signal from the aged APPswe mice was reduced, compared with that in the hippocampus from aged wild-type mice (Fig. 1D), suggesting an association of L1-70 and L1 with the occurrence or development of AD. Furthermore, we also explored the deposition of Aβ in the hippocampus from aged APPswe mice and aged wild-type mice by immunofluorescence staining with antibody against Aβ. We found that Aβ staining in the hippocampus from aged APPswe mice was more prominent than from aged wild-type mice (Fig. 1E). Together, these observations indicate a decrease of the L1-70 and L1 expression is concomitant with the deposition of Aβ in the hippocampus of the APPswe mouse but not in the wild-type mouse.

### L1-70 decreases Aβ deposition in the hippocampus of aged APPswe mice

To further explore a putative correlation of the decrease of L1-70 levels with an elevation of Aβ deposition in the brain of aged APPswe mice, we tested our hypothesis that molecules that exist in the blood of healthy young mice can promote L1-70 expression, which in turn minimizes Aβ deposition. We used the parabiosis approach [29] in which the circulatory systems of two individual animals are joined. To assess the efficiency of joined circulation in parabiotic mice, a 2-month-old CAG-EGFP C57BL/6 mouse that constitutively expresses green fluorescent protein (GFP) was joined to a 2-month-old wild-type C57BL/6 mouse. One week after parabiotic joining, GFP^+^ leukocytes were recovered from the blood of the wild-type mouse (40%) and amounted to 45% in the CAG-EGFP mouse (Supplementary Fig. 2).

**Fig. 2:**
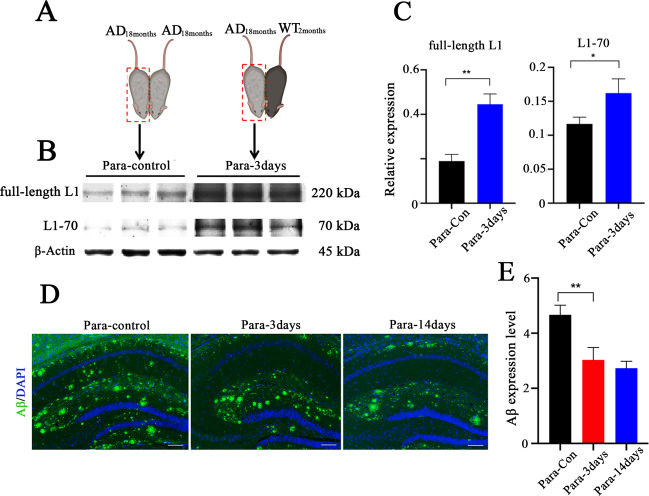
Expression of L1-70 and the deposition of Aβ in the hippocampus of aged APPswe mice in parabiosis with young wild-type mice. **A** Western blot analysis of hippocampal tissue homogenates from aged APPswe mice in parabiosis with same old APPswe mice as control (Para-control) (joined 3 days) and with 2-month-old young wild-type mice (joined 3 days), antibodies against the L1 cytoplasmic domain and against β-actin were used for western blot analysis. **B** Expression of L1-70 and L1 are shown. **C** Relative full-length L1 and L1-70 levels were calculated and normalized to β-actin. The data represent means ± SD, *p < 0.05, **p < 0.01, Student’s *t*-test, *n* = 3 mice in per group. **D** Representative immunofluorescence images of Aβ in the hippocampal tissue sections from aged APPswe mice in parabiosis with same old APPswe mice as control (joined 14 days) and with 2-month-old young wild-type mice (joined 3 days and 14 days). Antibody against Aβ was used for immunostaining. **E** Aβ expression levels were calculated using ImageJ. Data were shown as means ± SD, **p < 0.01, one-way ANOVA with Tukey’s post hoc test, *n* = 3 mice per group. Scale bars: 100 µm.

Having confirmed efficient blood exchange between parabiotically joined animals, the circulatory system of an aged (18 months old) APPswe mouse was joined to that of a healthy, young (2 months old) wild-type mouse, to assess whether circulatory components from the young mouse can affect L1/L1-70 expression and Aβ deposition in the aged APPswe mouse (Fig. 2A). Parabiosis of two aged APPswe mice served as the control group (Fig. 2B). In the aged APPswe to young wild-type pairing, the aged APPswe animal was assessed for L1-70 expression and for a degree of Aβ deposition. We observed elevated full-length L1 and L1-70 in the APPswe mouse within 3 days after parabiosis with a younger healthy mouse, compared to the control aged APPswe-APPswe group (Fig. 2C). Moreover, hippocampus deposition of Aβ was also reduced by Day 3 and with even more striking Aβ deposition reduction by Day 14. No changes in Aβ or in L1/L1-70 were observed in the control aged APPswe-APPswe pairings (Fig. 2D, E). Together, these observations point to the existence of serum factors in young healthy mice that can promote L1-70 expression and lead to decreased Aβ deposition in the aged APPswe animals.

### L1-70 combines with Top1 to form a complex that enters the nucleus of neural cells

To evaluate whether increasing L1-70 levels could contribute directly to reduce Aβ levels, we explored how L1-70 in the nucleus could regulate transcription. First, co-immunoprecipitation and mass spectrometry was performed to find candidate proteins that might associate with L1 from the 5- to 7-day-old wild-type mouse brain. Among the many proteins found, we focused on topoisomerase 1 (Top1), identified by mass spectrometry.

**Fig. 3:**
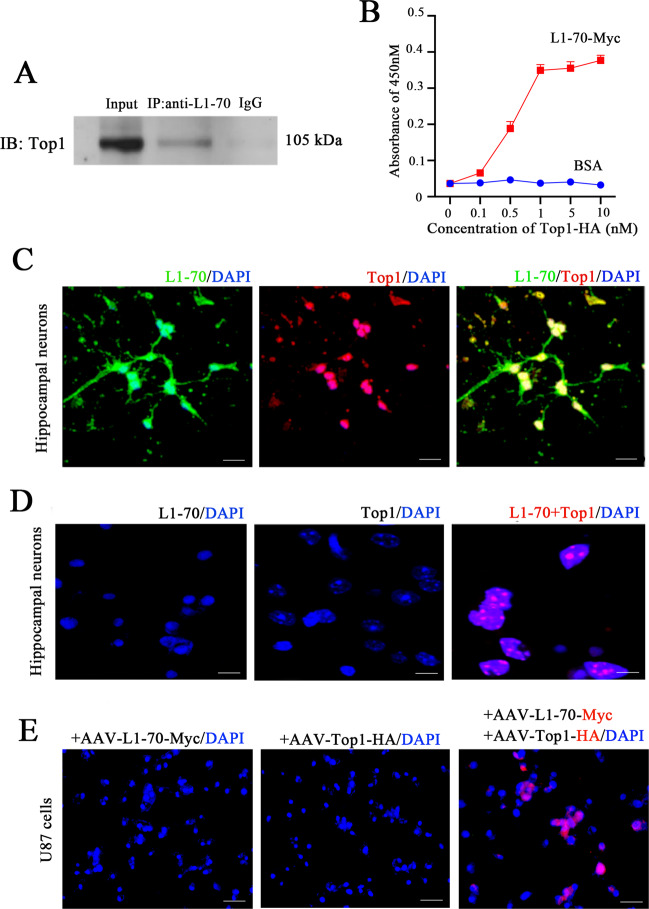
L1-70 is associated with Top1. **A** Co-immunoprecipitation of the hippocampus tissue homogenates from 5- to 7-day-old wild-type mice with an antibody against L1 cytoplasmic domain being used for the co-immunoprecipitation and antibody against Top1 being used for western blotting. **B** ELISA for measuring the interaction between L1-70 and Top1 which were prepared from genetically engineered bacteria expressing recombinant L1-70-Myc and Top1-HA. Antibodies against Myc and HA tags were used for the identification of L1-70 protein and Top1 protein. The absorbance of L1-70-myc increased with the higher concentration of Top1-HA and showed saturation at 1 nM. **C** Co-expression of L1-70 and Top1 in primary cultured cells from the hippocampus of wild-type newborn mice. Immunofluorescence staining for L1-70 and Top1. L1-70 (green) colocalized with Top1 (red) in the nucleus. **D** Proximity ligation assay (Duolink) of the interaction between endogenous L1-70 protein and Top1 protein in primary hippocampal neurons from wild-type newborn mice. L1-70/Top1 complexes are indicated by the red dots. **E** Proximity ligation assay (Duolink) of the interaction between recombinant L1-70-Myc protein and Top1-HA protein transduced in U87 cells (which lack expression of L1) with recombinant adeno-associated virus expressing L1-70-Myc or/and Top1-HA. Antibodies against Myc and HA tags were used in the Duolink analysis. Three groups were transduced with recombinant viruses, AAV-Top1-HA, AAV-L1-70-Myc, or both. Positive signals could be found only in U87 cells transduced with both adeno-associated viruses. Scale bar: 20 µm.

In order to further explore the L1-70/Top1 interaction, immunoprecipitation was performed with brain lysates using an antibody against the L1 cytoplasmic domain and probing the immunoprecipitate with an anti-Top1 antibody by western blot analysis (Fig. 3A). In the input and IP groups, no band was seen in the nonimmune IgG control group (Fig. 3A). These results indicate that L1-70 can associate with Top1 in the wild-type brain.

To further confirm that L1-70 binds to Top1 directly, we transfected 293 T cells with vectors expressing a myc-tagged L1-70 (*CMV-L1-70-Myc*) and HA-tagged Top1 (*CMV-Top1-HA*), and the binding of L1-70 and Top1 was analyzed by ELISA using plates coated with antibodies against either Myc or HA. In this experiment, purified L1-70-Myc from transfected 293 T cells was added to the substrate-coated target molecules. Then, a series of dilutions of purified Top1-HA was added to allow binding to L1-70. The results showed that L1-70 binds to Top1 in a concentration-dependent manner (Fig. 3B), confirming that L1-70 and Top1 bind directly to each other.

Next, we assessed the localization of L1-70/Top1 complexes in hippocampal cultures by double immunofluorescence staining with antibodies against L1 and Top1. Positive staining of L1-70 (green) and Top1 (red) were observed to overlap in the nucleus (Fig. 3C), suggesting an association between the molecules. Then, proximity ligation (Duolink) was performed to analyze the possibility that the association was within a distance of 40 nm or less. Positive Duolink amplification signals (red) were detected in many nuclei (Fig. 3D), confirming that nuclear L1 and Top1 exist as an endogenous complex.

To verify the binding of exogenous L1-70-myc and Top-HA, cultured hippocampal cells were transduced with adeno-associated viruses expressing L1-70-Myc and Top-HA (*AAV-L1-70-Myc* and *AAV-Top1-HA*, Supplementary Fig. 3), and a proximity ligation assay (PLA) was performed with antibodies against Myc and HA. Positive reaction signals (red) appeared in the nuclei of cultured hippocampal cells (Fig. 3E), indicating binding of L1-70 and Top1 to each other. Taken together, these results demonstrate that L1-70 combines with Top1 to form an L1-70/Top1 nuclear complex.

### L1-70/Top1 complexes regulate the expression of macrophage migration inhibitory factor (MIF) in neural cells

**Fig. 4:**
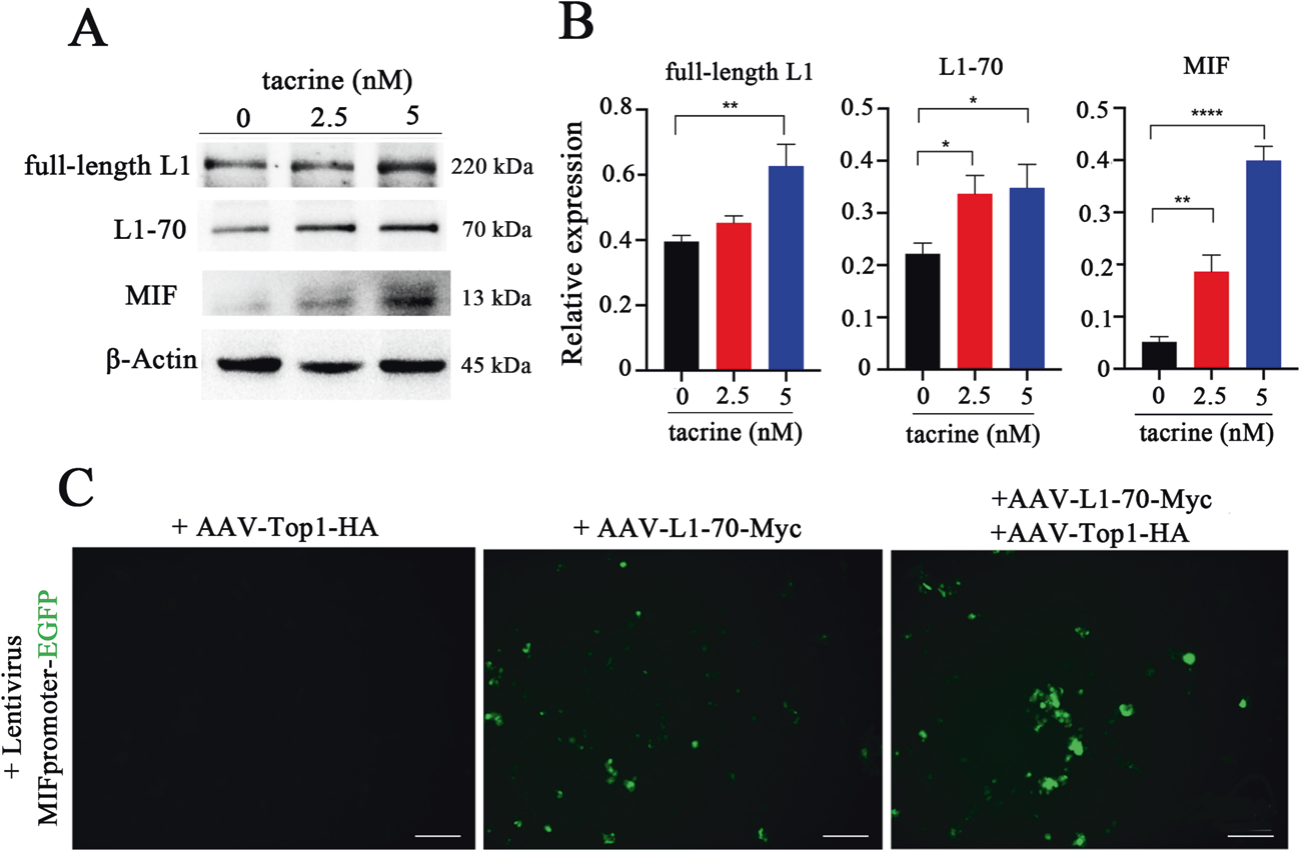
The L1-70/Top1 complex regulates MIF expression in hippocampal neurons. **A** Western blot analysis of the cell lysates of mouse neuroblastoma N2a cells treated with tacrine. Antibodies against L1 cytoplasmic domain, against MIF and against β-actin were used for immunoblotting. Expression of L1 and L1-70 was induced by tacrine and accompanied by an increase in MIF expression level. **B** Relative full-length L1, L1-70, and MIF levels were calculated and normalized to β-actin. The data represent means ± SD, *p < 0.05, **p < 0.01, ****p < 0.0001, one-way ANOVA with Tukey’s post hoc test, three independent experiments. **C** U87 cells were transduced with lentivirus, encoding a MIF promoter-driven EGFP, for 48 h. Then, *AAV-L1-70-Myc*, *AAV-Top1-HA*, and both *AAV-L1-70-Myc* and *AAV-Top1-HA* were added. After 36 h, a strong EGFP signal could be found in cells transduced with both *AAV-L1-70-Myc* and *AAV-Top1-HA*.

Next, we asked whether the L1/Top1 complex can act as a transcription factor to regulate gene expression in neurons. Chromatin immunoprecipitation was performed to identify candidate target genes. To this aim, the chromatin of cultured wild-type hippocampal neurons was sheared by ultrasound into fragments around 250 bp, and the DNA fragments bound to L1-70 were isolated by pull-down with antibody against L1-70. Among a large number of candidate genes, we selected macrophage migration inhibitory factor (MIF) because it had been reported to activate microglial cells and take part in the clearance of Aβ [30–33]. To stimulate the association of L1-70 with Top1 to regulate MIF gene expression, the agonistic L1 mimetic tacrine was used to induce high expression of L1 [34] in mouse neuroblastoma N2a cells. We found that the expression level of L1-70 and L1 was increased when N2a cells were treated with increasing concentrations of tacrine (0, 2.5, and 5 nM) (Fig. 4A, B).

Next, we constructed a lentivirus expression system, in which the MIF promoter drives the expression of EGFP (*pMIF-EGFP*). We also engineered two adeno-associated viruses to express L1-70-Myc and Top1-HA. Cultured human glioblastoma U87 cells, which normally do not express L1, were transduced with lentivirus, encoding the recombinant *MIF-EGFP* gene, for 48 h, then the cells were further transduced with adeno-associated virus mediating the expression of L1-70-Myc and Top1-HA for an additional 24 h. The *EGFP* gene was expressed strongly in U87 cells transduced with both *AAV-L1-70-Myc* and *AAV-Top1-HA* genes compared to no signal following transduction with *AAV-Top1-HA* only and a low signal transduced with *AAV-L1-70-Myc* only (Fig. 4C). This observation indicates that the expression of MIF is regulated by the L1/Top1 complex.

### MIF activates microglia in the hippocampus of aged APPswe mice and facilitates the reduction of amyloid plaques

**Fig. 5:**
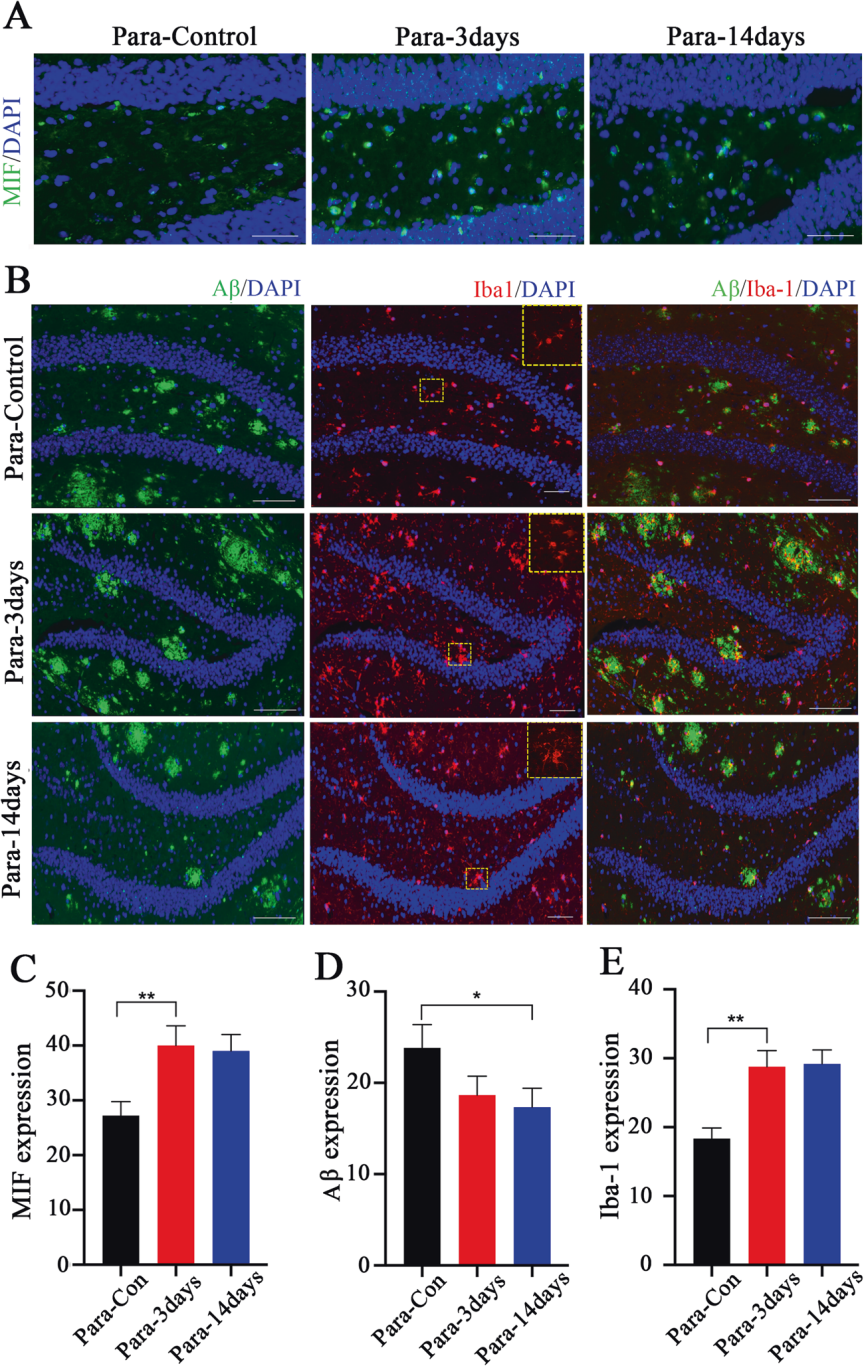
MIF expression correlates with Iba-1 expression and Aβ clearance. **A** Representative immunofluorescence staining of hippocampal tissue sections from aged APPswe mice in parabiosis with aged APPswe mice. Antibody against MIF was used for staining. Compared to the Para-control group (two 18-month-old APPswe mice joined together), more positive signals appeared in APPswe mice in the APPswe-young wild-type parabiosis group at 3 and 14 days. **B** Representative immunofluorescence staining for hippocampal tissue sections from aged APPswe mice in parabiosis with aged APPswe mice or young wild-type mice for 3 days and for 14 days. More Iba-1 appeared in the 3-day group compared to the control, suggesting more microglia were activated to cluster around Aβ. Antibodies against Iba-1 and Aβ were used for staining. **C**–**E** MIF, Aβ, and Iba-1 expression levels were calculated using ImageJ. Data were shown as means ± SD, *p < 0.05, **p < 0.01, one-way ANOVA with Tukey’s post hoc test, *n* = 3 mice per group. Scale bars: **A** 50 µm, **B** 100 µm.

Based on our finding that Aβ deposition is reduced in the hippocampi of aged APPswe mice by parabiosis with young wild-type mice, we examined the possibility that microglia are involved in this process. Paraffin sections of hippocampal tissues of aged APPswe mice were analyzed by immunofluorescence with antibodies against MIF, Iba-1, and Aβ. Expression of MIF was increased following a 3-day parabiosis of APPswe mice with young wild-type mice (Fig. 5A, C). Furthermore, in the 14-day parabiosis group, the amyloid plaques were reduced (Fig. 5B, D). Moreover, the number of microglia in the dentate gyrus of the aged APPswe mice was increased compared to the control group (Fig. 5B, E). Microglia were also in an activated state and clustered around the amyloid plaques. Based on these findings and the known role of the microglia in the clearance of Aβ deposition, activation of microglia-mediated by MIF is likely to play a role in the reduction of Aβ in the AD brain.

### Blood TGFβ-1 mediates the expression of L1-70 in neurons

**Fig. 6:**
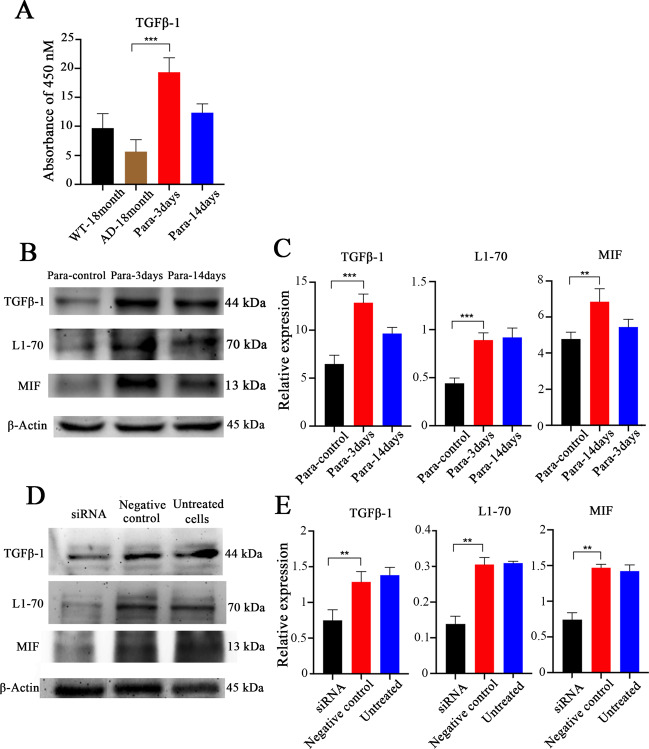
Correlation of TGFβ-1 expression with L1-70 and MIF expression. **A** ELISA analysis of TGFβ-1 in the blood of aged wild-type mice, aged APPswe mice, and aged APPswe mice in parabiosis with young wild-type mice for 3 and 14 days. **B** Western blot analysis of hippocampal tissue homogenates from aged APPswe mice in parabiosis with aged APPswe mice, and aged APPswe mice in parabiosis with young wild-type mice for 3 days and 14 days. Antibodies against TGFβ-1, L1 cytoplasmic domain, MIF, and β-actin were used for immunoblotting. **C** Relative to β-actin levels more TGFβ-1, L1-70, and MIF are seen on day 3 and day 14 compared to the control group. The data represent means ± SD, **p < 0.01, ***p < 0.001, one-way ANOVA with Tukey’s post hoc test, three independent experiments. **D** Western blot analysis of cell lysates of cultured N2a cells in which the expression of TGFβ-1 gene was knocked down with siRNA. Antibodies against TGFβ-1, L1 cytoplasmic domain, and MIF were used for immunoblotting. The expression of L1-70 and MIF decreased with the knockdown of TGFβ-1. **E** Relative TGFβ-1, L1-70, and MIF levels were calculated and normalized to β-actin. The data represent means ± SD, **p < 0.01, ***p < 0.001, one-way ANOVA with Tukey’s post hoc test, three independent experiments.

Since the blood from young mice increases MIF expression in the hippocampus of aged APPswe mice and could activate microglia to eliminate Aβ, we asked which factor(s) in the blood from young mice are involved in this process. It has been shown that the expression of L1 in pancreatic duct cells and intestinal epithelial cells can be induced by TGFβ-1 [35–37]. Therefore, we investigated the relationship between TGFβ-1 in the blood and the expression of L1-70 and MIF in the hippocampus of aged APPswe mice. First, TGFβ-1 was determined by ELISA in the sera from aged wild-type mice, aged APPswe mice, and aged APPswe mice after a 3- and 14-day-long parabiosis of aged APPswe mice with young wild-type mice. The amount of TGFβ-1 in the sera of aged APPswe mice was lower than that in the sera of aged wild-type mice. However, the TGFβ-1 level in aged APPswe mice in parabiosis with young wild-type mice was higher than in the sera from aged wild-type mice and aged APPswe mice without parabiosis (Fig. 6A). Serum TGFβ-1 was highest in aged APPswe mice that had undergone parabiosis for 3 days. Furthermore, we used western blotting to analyze the relationship of TGFβ-1 with the expression of L1-70 and MIF in hippocampi of aged APPswe mice from the 3-day parabiosis of aged APPswe mice with aged APPswe mice, and the 3- and 14-day-long parabiosis of aged APPswe mice with young wild-type mice. Serum TGFβ-1 levels paralleled L1-70 and MIF levels (Fig. 6B, C), supporting the view that circulating TGFβ-1 could regulate L1-70 and MIF in the brain.

In order to further investigate the relationship between circulating TGFβ-1, L1-70, and MIF expression, mouse neuroblastoma N2a cells were transfected with TGFβ-1 siRNA. After 48 h, decreased TGFβ-1 expression in the siRNA groups was accompanied by a decrease in L1-70 and MIF expression (Fig. 6D, E), further supporting the notion that the function of TGFβ-1 is associated with the expressions of L1-70 and MIF.

## Discussion

Alzheimer’s disease was recognized as early as in 1901. In the 1960s, the “amyloid cascade hypothesis” and “tau protein hyperphosphorylation hypothesis” were proposed to explain AD pathogenesis, promoting the rapid development of AD research. However, these efforts have been unsuccessful in developing effective drugs for AD remission or cure. This failure points to the complexity of AD pathogenesis, highlighting the limits of the current understanding of AD pathogenesis [38, 39].

Cell adhesion molecule L1 has emerged to be interesting in dealing with the pathophysiology of AD since it has been shown to ameliorate the consequences in several mouse models of neurodegenerative diseases [18]. It is interesting in this respect that the extracellular domain of L1 can be associated with several ligands, such as other members of the Ig superfamily, integrins or extracellular matrix proteins [40–42]. Also, the intracellular domain can mediate the functional connection between the actin cytoskeleton and endoplasmic trafficking system, thus participating in axonal targeting, cell surface stability, and synaptic activities [43, 44]. Moreover, L1 is involved in establishing and changing connections between neurons in the traumatized/regenerating nervous system and in synaptic plasticity [45, 46]. Under certain physiological and pathological conditions, full-length L1 can be cleaved by proteolysis resulting in several proteolytic fragments, among them L1-70, containing the complete intracellular domain and part of the extracellular domain [28]. The L1-70 can enter the cytoplasm by endocytosis and enter mitochondria and nuclei [25, 26].

We reported an increase of L1 in the cerebrospinal fluid of AD patients [19] and also the reduction of amyloid plaques in the hippocampus and occipital cortex of AD mice following transduction with L1-expressing adeno-associated virus and recombinant L1. L1 was found to bind to Aβ but not to APP [20]. These studies suggest that the occurrence and development of AD may be related to the abnormal regulation of L1 in neural cell functions.

We have extended these studies to show the following: expression of L1 and L1-70 in the brain tissue of aged AD mice is lower in comparison to wild-type mice. Decreased L1-70 expression in these animals is accompanied by more amyloid plaques. In contrast, high levels of L1-70 in the wild-type hippocampus are accompanied by the absence of amyloid plaques. Using a parabiosis approach, in which the blood circulation of aged AD mice is joined with the blood circulation of young wild-type mice, we show that L1-70 expression increases within 3 days, and that amyloid plaques decrease within 14 days. These results suggest that L1-70 is involved in regulating the clearance of amyloid plaques. We also show that L1-70 forms a complex with Top1 in the nucleus where it may regulate transcription. As shown by ChIP with anti-L1 antibody and DNA sequencing to identify DNA fragments specifically binding to the L1-70/Top1 complex, and using MIF reporter gene assays, we showed that the L1-70/Top1 complex regulates MIF expression, which can activate microglia and promote clearance of Aβ in the brain [31, 32]. Furthermore, the agonistic L1 mimetic tacrine increased L1-70 and MIF expression, further supporting the notion that the L1-70/Top1 complex regulates MIF expression. MIF expression was increased in the brain of AD mice following parabiosis with young wild-type mice, while reducing amyloid plaques, suggesting that MIF indirectly reduces Aβ deposition in the AD brain via L1-70. Notably, high numbers of microglial cells were observed in the vicinity of amyloid plaques. These results support the view that MIF may eliminate Aβ through the activation of microglia.

In pancreatic duct cells, TGFβ-1 had been shown to upregulate the expression of L1 [35]. In our parabiosis experiments, the transfer of TGFβ-1 from young mice to aged AD mice could regulate the expression of L1-70. To investigate whether TGFβ-1 could upregulate the expression of L1, TGFβ-1 levels in the serum were measured, and TGFβ-1 siRNA was applied to cultured neuroblastoma cells. We found that TGFβ-1, L1-70, and MIF were increased in the serum of old AD mice at 3 days after parabiosis surgery, and knock down of TGFβ-1 with siRNA in neuroblastoma cells decreased L1-70 and MIF expression along with the decrease in TGFβ-1. These results suggested that TGFβ-1 could be transferred through the blood circulation to upregulate L1-70 expression in AD mice.

**Fig. 7:**
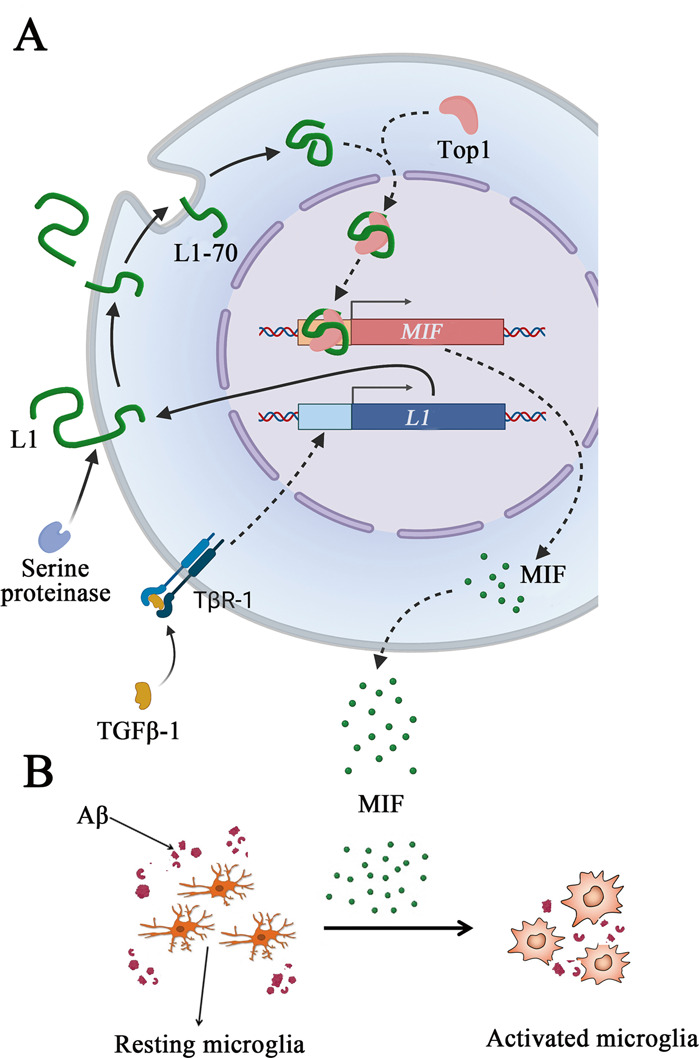
Working model of L1-70 involved in the clearance of Aβ in the brain. **A** Under the action of serine-dependent proteases, the L1-70 can be cleaved from full-length L1 molecule located on the plasma membrane of neural cells, and then since the evidence for L1-70 binding to Top1 in the nucleus to form a complex which can regulate the expression of MIF gene. **B** MIF could then be secreted from the cells to activate microglia and promote the clearance of Aβ in the brain.

In conclusion, using a mouse model of AD, we found a correlation between L1-70 levels and reduction of amyloid plaques in a mouse model of AD. The data suggest that L1-70 is an intermediate molecule involved in Aβ clearance (Fig. 7). These observations point to the need for further studies: for example, by which mechanism is L1-70 production regulated in the normal brain? Why is the expression level of L1-70 decreased in the brains of aged AD mice? What is the mechanism by which TGFβ-1 regulates the production of L1-70? Can exogenous TGFβ-1 reduce Aβ accumulation or remove the deposited Aβ plaques? We expect that the present study has yielded novel insights into the understanding of AD pathogenesis.

## Materials and methods

### Animals

Wild-type C57BL/6 mice were purchased from the Guangdong Medical Laboratory Animal Center (Foshan, China), APPswe transgenic mice were purchased from the Model Animal Research Center of Nanjing University (Nanjing, China). All animals were bred in the Center of Experimental Animals of Shantou University Medical College. Animal experiments were in accordance with the guidelines approved by the Institutional Committee of Shantou University according to international standards. All efforts and attention were given to minimize the pain and the number of mice used for the experiments. The mice were selected randomly for the experiments.

### Antibodies and reagents

The following antibodies and reagents were used in the experiments: mouse anti-L1 cytoplasmic domain (Biolegend, catalog # 838101), mouse anti-β-amyloid (Abcam, catalog # ab126649), mouse anti-MIF (Santa Cruz Biotechnology, catalog # sc-271631), rabbit anti-topoisomerase I (Novus Biologicals, catalog # NBP1-90365), rabbit anti-Iba-1 (Wako, catalog # 015-28011), rabbit anti-TGFβ 1 (Proteintech, catalog # 21898-1-AP), mouse anti-β-actin (Boster, catalog # BM0627), mouse anti-Myc (Boster, catalog # BM0238), and rabbit anti-HA (Bioworld Technology, catalog # AP0005). Secondary antibodies coupled to horseradish peroxidase or fluorescein were: goat anti-mouse (Boster, catalog # BA1051), goat anti-rabbit (Boster, catalog # BA1055), Alexa Fluor 488-conjugated donkey anti-mouse (ImmunoResearch, catalog # A21202), Alexa Fluor 594-conjugated donkey anti-rabbit (ImmunoResearch, catalog # A10042). Reagents and kits were radioimmunoprecipitation assay (RIPA, Solarbio, catalog # R0010), phenylmethanesulfonyl fluoride (PMSF, Solarbio, catalog # IP0280), LDS sample buffer (Thermo Fisher, catalog # B0008), sample reducing agent (Thermo Fisher, catalog # B0004), BCA protein assay kit (Solarbio, catalog # PC0020), PVDF membrane (Millipore, catalog # 3010040001), enhanced chemiluminescence kit (ECL, Beyotime, catalog # P0018FS), TMB substrate solution for ELISA (Beyotime, catalog # P0209-100), Lipofectamine 2000 (Invitrogen, catalog # 11668027), Co-IP kit (Thermo Fisher, catalog # 26149), Duolink kit (Sigma, catalog # DUO92101), ChIP kit (Merck Millipore, catalog # 16-662), protease inhibitor cocktail (Sigma, catalog # P1860), tacrine (Med Chem Express, catalog # HY-B1488). ProLong^®^ Gold Antifade reagent with DAPI (Invitrogen, catalog # P36935) was used to stain cell nuclei. Cell culture dishes were coated with poly-d-lysine (Beyotime, catalog # ST508). Adeno-associated virus and lentivirus were constructed by VectorBuilder.

### Western blot analysis

Western blot analysis was performed as described by Sahu et al. (2016). Total proteins were collected by extracting brains tissues and cells in RIPA buffer supplemented with PMSF, mixed with 25% SDS-PAGE loading buffer, and then heated for 10 min at 100 °C. Forty micrograms of each protein sample was subjected to 10% SDS-PAGE and transferred onto PVDF membranes. After blocking with 5% BSA, the blots were probed with the following primary antibodies overnight at 4 °C: mouse anti-L1 cytoplasmic domain (1:1000, Biolegend, catalog # 838101), rabbit anti-topoisomerase I (1:1000, Novus Biologicals, catalog # NBP1-90365), mouse anti-MIF (1:1000, Santa Cruz Biotechnology, catalog # sc-271631), rabbit anti-TGFβ-1 (1:1000, Proteintech, catalog # 21898-1-AP), and mouse anti-β-actin (1:1000, Boster, catalog # BM0627). Membranes were then washed three times with PBST (phosphate-buffered saline, pH 7.3, containing 0.1% Tween-20) for 5 min each wash, followed by incubation with secondary goat anti-mouse (1:2000, Boster, catalog # BA1051) and goat anti-rabbit (1:2000, Boster, catalog # BA1055) in 5% BSA for 2 h at room temperature (RT). Membranes were then washed three times in TBST for 5 min each wash at RT. Bands were visualized with an enhanced chemiluminescence kit (ECL, Beyotime, catalog # P0018FS). Signal intensity was detected and quantified using ImageJ software (National Institutes of Health).

### Culture of mouse hippocampal neurons

Hippocampal neurons from 1-day-old wild-type C57BL/6 mice were cultured. In brief, cell culture dishes were coated with poly-d-lysine. Tissues were digested with trypsin in Hank’s balanced salt solution and gently triturated. After filtration and centrifugation, cells were resuspended in DMEM/F-12 culture medium (HyCloneTM, catalog # 10565018) with 10% fetal bovine serum (Every Green, catalog # 11011-8611). Cell suspensions were seeded onto poly-d-lysine-coated 48-well or 24-well plates at 5 × 10^4^ and 1 × 10^5^ cells in DMEM/F-12 (HyCloneTM, catalog # 10565018) per well, respectively. After 4–6 h, the medium was removed, and Neurobasal-A medium (Thermo Fisher # 10888022) supplemented with 2% B-27 (Thermo Fisher # 17504044) and 1% penicillin-streptomycin solution (Solarbio # P1400) was added, and cells were maintained at 37 °C in a 5% CO_2_ atmosphere.

### Immunofluorescence staining

Cultures of hippocampal neurons were fixed with 4% formaldehyde solution for 10 min at RT and washed with phosphate-buffered saline, pH 7.3 (PBS) three times for 5 min each wash. Then, 0.1% Triton X-100 in PBS was used to permeabilize the plasma membrane. Hippocampal sections were selected randomly and fully dewaxed, and antigen retrieval was performed in citrate buffer (0.01 M, pH 6.0) for 40 min at 99 °C. To block nonspecific binding sites, cells and sections were incubated with PBS containing 10% normal donkey serum for 1 h at RT. Samples were then incubated at 4 °C overnight with mouse anti-L1 cytoplasmic domain (1:400, Biolegend, catalog # 838101), rabbit anti-topoisomerase I (1:400, Novus Biologicals, catalog # NBP1-90365), mouse anti-β-amyloid (1:400, catalog # ab126649), or rabbit anti-Iba-1 (1:400, Wako, catalog # 015-28011). After washing with PBS three times for 5 min at RT, samples were incubated for 2 h at RT with donkey anti-mouse secondary antibody conjugated to DylightTM 488 (1:500, ImmunoResearch, catalog # A21202) or donkey anti-rabbit secondary antibody conjugated to DylightTM 594 (1:500, ImmunoResearch, catalog # A10042). After three 5-min washes with PBS at RT, sections or cells were mounted using ProLong^®^ Gold Antifade reagent with DAPI (Invitrogen, catalog # P36935). The intensity of immunopositivity in cultured neurons was collected using a fluorescence microscope (Axio Imager Z1, Zeiss). Fluorescence images from tissue sections were acquired with an Olympus laser confocal system (FV-1000, Olympus).

### Parabiosis surgery

Parabiosis is the surgical union of two related organisms (e.g., young and old) allowing sharing of the blood circulation and neovascularization without triggering an immunological reaction. The procedure was performed according to Paniz [29]. Two-month-old wild-type C57BL/6 mice were partnered with 18-month-old APPswe transgenic mice (*n* = 6 mice per group). After partnering for 3 or 14 days, the hippocampus from the APPswe mice was prepared for immunofluorescence staining and western blotting at 3 and 14 days, respectively.

### Co-immunoprecipitation

Co-immunoprecipitation was performed following the company’s instructions (Thermo Fisher, catalog # 26149). In brief, mouse anti-L1 cytoplasmic domain (Biolegend, catalog # 838101), or nonimmune mouse IgG antibody as control were incubated with beads for 2 h at RT. Wild-type C57BL/6 mouse (5–7 days old) brain lysates were incubated with antibody-bound beads overnight at 4 °C. After washing in IP buffer (0.025 M Tris, 0.15 M NaCl, 0.001 M EDTA, 1% NP-40, 5% glycerol; pH 7.4) and conditioning buffer (neutral pH buffer), the beads were washed and the protein complexes bound to the antibody were eluted with elution buffer (contains primary amine, pH 2.8). Subsequent western blot analyses were performed as described.

### ELISA

The same concentration of purified L1-70-Myc in PBS was used for substrate coating in 96-well plates at 4 °C overnight. After five 10-min washes in PBST (phosphate-buffered saline, pH 7.3, containing 0.1% Tween-20) at RT, wells were blocked with 10% fetal bovine serum (Every Green, catalog # 11011-8611) in PBS for 3 h at 37 °C, then washed five times with PBST, 10 min each wash at RT. Several concentrations (0.1, 0.5, 1, 5, and 10 nM) of purified protein pCMV-Top1-HA were added to each well and incubated for 2 h at 37 °C. After washing with PBST, the wells were incubated with rabbit anti-HA for 1.5 h at 37 °C. OD values were determined using TMB (3, 3′,5,5′-tetramethylbenzidine) substrate solution for ELISA (Beyotime, catalog # P0209-100).

### Proximity ligation assay

In order to investigate the relationship between L1 and Top1, a proximity ligation assay (Duolink) was performed. Duolink (Sigma, catalog # DUO92101) provides a sensitive method for in situ detection of proteins that co-localize at a distance of 40 nm or less. The experiment was performed according to the manufacturer’s instructions. Hippocampal neurons were prepared in the 24-well plates, and a blocking buffer was added to saturate nonspecific binding sites. Neurons were incubated with mouse anti-L1 cytoplasmic domain (Biolegend, catalog # 838101) and rabbit anti-Top1 (Novus Biologicals, catalog # NBP1-90365) antibodies at 4 °C overnight. Neurons incubated with only one antibody were taken as the negative control. After washing with Buffer A, the secondary antibodies with specific anti-mouse and anti-rabbit PLA probes were added and neurons were incubated at 37 °C for 1 h. Subsequently, the reaction solution for the ligase was added and incubated at 37 °C for 30 min, resulting in the circularization of the two adjacent PLA probes. Reaction amplifiers with polymerase were added and incubated at 37 °C for 100 min to visualize the red dots. Before imaging, slides were washed with Buffer A and mounted on a coverslip using Duolink In Situ Mounting Medium with DAPI. Confocal images were acquired with an Olympus laser confocal microscope (Olympus, FV-1000).

### Lentiviral transduction to determine the regulation of MIF expression by L1-70 and Top1

The adeno-associated virus and lentivirus were constructed by VectorBuilder (constructions in the Supplemental Fig. 3). U87 human glioblastoma cells were transduced with lentivirus encoding an EGFP driven by the MIF promoter. After 48 h, transduced cells were infected with adeno-associated virus encoding L1-70-Myc and adeno-associated virus encoding Top1-HA. To characterize the expression of L1-70-Myc and Top1-HA, western blotting was performed with the primary antibodies against Myc (Boster, catalog # BM0238) and against HA (Bioworld Technology, catalog # AP0005). To investigate the formation of L1-70/Top1 complexes, Duolink was performed following the provider’s instructions (Sigma, catalog # DUO92101).

### Chromatin immunoprecipitation (ChIP)

Hippocampal neurons were prepared for ChIP assay following the manufacturer’s protocol (Merck Millipore, catalog # 16-662). Cross-linking between protein and chromatin was achieved by adding 1% formaldehyde in PBS for 15 min at RT, followed by quenching with 125 mM glycine for 5 min at RT. Cells were harvested in PBS and lysed with immunoprecipitation (IP) buffer containing 150 mM NaCl, 50 mM Tris-HCl (pH 7.5), 5 mM EDTA, Nonidet P-40, and Triton X-100, supplemented with protease inhibitor cocktail (Sigma, catalog # P1860). Nuclear pellets were isolated by centrifugation at 12,000x*g* for 1 min and resuspended in an IP buffer. The chromatin then was cleaved by sonication on ice. For isolation of L1-bound complexes, the chromatin solution was incubated with anti-L1 cytoplasmic domain (Biolegend, catalog # 838101) overnight at 4 °C. Nonimmune mouse IgG was used for control. After IP, cross-linking was reversed by boiling the sample with Chelex100 (Bio-Rad, catalog # 1421253) for 10 min. The supernatant containing DNA fragments was isolated by centrifugation at 12,000x*g* for 1 min.

### N2a cell culture and tacrine treatment

The L1 agonistic mimetic tacrine upregulates the expression of L1 in cultured L1-expressing cells [23]. N2a cells were placed in a 6-well plate at 1.2 × 10^6^ cells per well for 24 h. Tacrine dissolved in Dulbecco’s modified Eagle’s medium F-12 (HyCloneTM, catalog # 10565018) at different concentrations (0, 2.5, 5 nM) was added to the wells. After culturing for 48 h, cells were collected in RIPA buffer (Solarbio, catalog # R0010) supplemented with PMSF (Solarbio, catalog # IP0280), mixed with 25% sample loading buffer (Thermo Fisher, catalog # B0008), and heated at 100 °C for 15 min for western blot analysis.

### siRNA transfection

N2a cells were transfected with siRNA for TGFβ-1 (TGFβ-1 siRNA sense, 5′-GCUCUUGUGACAGCAAAGAUATT-3′; antisense, 5′-UAUCUUUGCUGUCACAAGAGCTT-3′) in a 6-well-plate by application of 50 nM siRNA or 50 nM control siRNA, using a Lipofectamine 2000 kit (Invitrogen, catalog # 11668027). Dulbecco’s modified Eagle’s medium F-12 (HyCloneTM, catalog # 10565018) was changed after 4–6 h and then cells were incubated for 48–72 h. Total protein was harvested, and the concentration was determined by a bicinchoninic acid test (Solarbio, catalog # PC0020) for western blot analysis.

### Statistical analyses

Statistical analyses were performed using SPSS 19.0 software (SPSS, Chicago, IL, USA). All values are presented as means ± standard deviation (SD). Student’s *t-*test was used to compare two treatment groups, and one-way analysis of variance (ANOVA) with Tukey’s post hoc test was used for multiple group comparisons. The variance was similar between the groups. Values of *p < 0.05, **p < 0.01, ****p < 0.0001 were considered statistically significant.

## Supplementary information

Legends of supplementary figure 1, 2 and 3

Supplementary Figure 1

Supplementary Figure 2

Supplementary Figure 3

## Data Availability

The datasets generated for this study are available upon reasonable request from the corresponding author.
