# Supplementary material for: A fragment of cell adhesion molecule L1 reduces amyloid-β plaques in a mouse model of Alzheimer’s disease
Source: Cell Death Dis. 2022 Jan 10;13(1):48. doi: 10.1038/s41419-021-04348-6 (PMC8748658; doi:10.1038/s41419-021-04348-6)
Supplement: Supplementary file 1 — Legends of supplementary figure 1, 2 and 3 [file 41419_2021_4348_MOESM1_ESM.doc]

**Supplementary Figure 1. Schematic diagram of L1-70 generation by proteolytic cleavage of full-length L1.** L1-70, a fragment with an apparent molecular weight of 70 kDa, can be generated by proteolytic cleavage of full-length L1 (220 kDa) by a serine-dependent protease.

**Supplementary Figure 2. Parabiosis model established between two individual mice. A.** The schematic diagram depicting the parabiosis model. **B.** Parabiosis surgery for the C57BL/6 (GFP^-^) mouse with the CAG-EGFP wild-type (GFP^+^) mouse, with the joined mice living together for one week. **C.** Flow cytometry analysis for the blood of joined mice. In the C57BL/6 mouse 40% of all cells are GFP^+^, suggesting that the two mice share their blood systems.

**Supplementary Figure 3. Schematic diagram of recombinant gene structures. A.** Structure of the recombinant reporter gene for the *MIF* gene. The coding sequence of *EGFP* is driven by the promoter of *MIF* gene, which was inserted into the cloning site of lentiviral expression vector, and then packaged into virus-like particles for cell transfection. **B.** Structure of recombinant *Top1* gene. The coding sequence of the *Top1* gene with nuclear sequence is driven by the CMV promoter, which was inserted into the cloning site of an adeno-associated virus expression vector, and then packaged into virus-like particles for cell transfection. **C.** Structure of recombinant *L1* gene. The coding sequence of the *L1* gene is driven by the CMV promoter, which was inserted into the cloning site of an adeno-associated virus expression vector, and then packaged into virus-like particles for cell transfection.
