# Supplementary figures and images for: A fragment of cell adhesion molecule L1 reduces amyloid-β plaques in a mouse model of Alzheimer’s disease
Source: Cell Death Dis. 2022 Jan 10;13(1):48. doi: 10.1038/s41419-021-04348-6 (PMC8748658; doi:10.1038/s41419-021-04348-6)

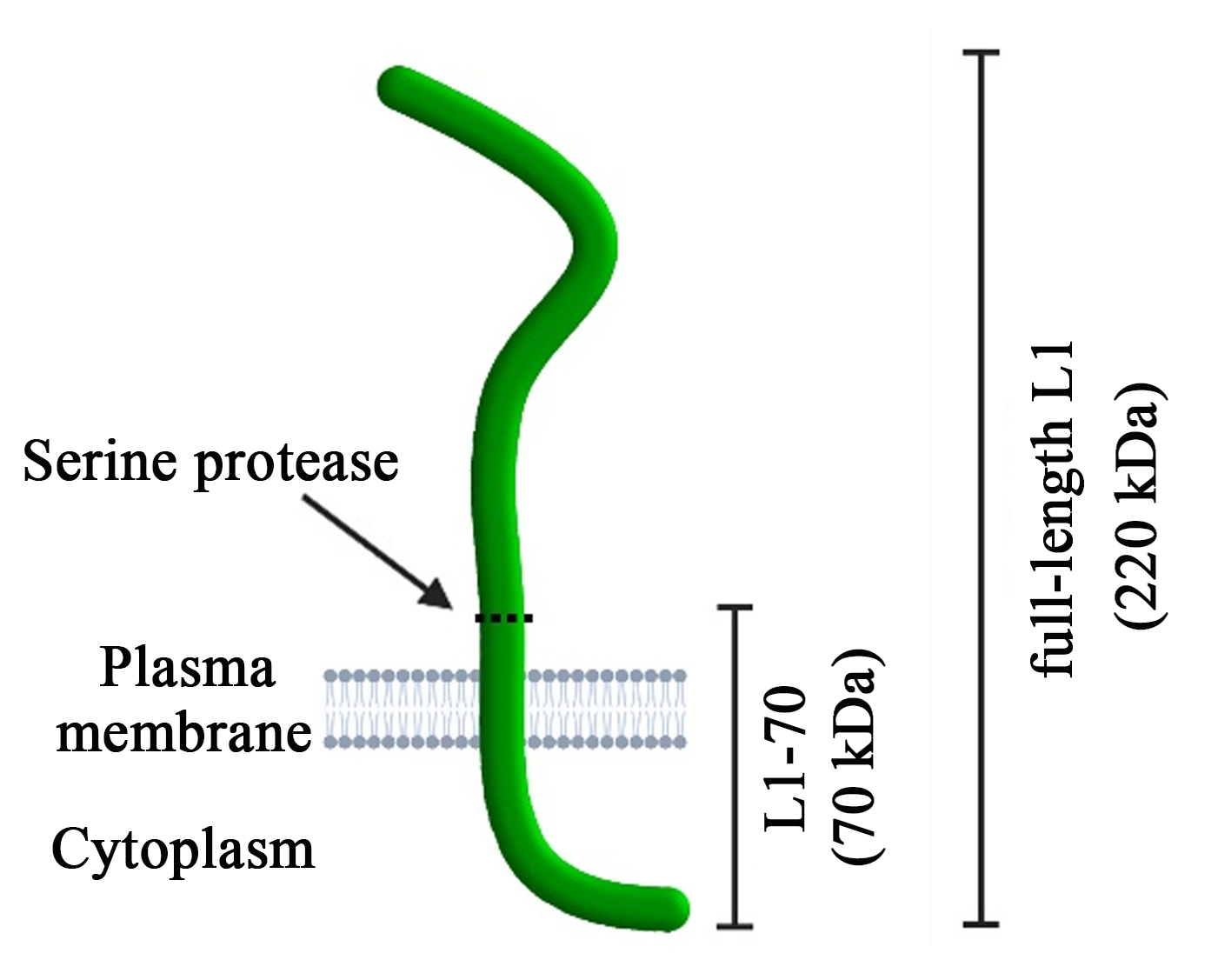

Supplement: Supplementary file 2 — Supplementary Figure 1 [file 41419_2021_4348_MOESM2_ESM.tif]

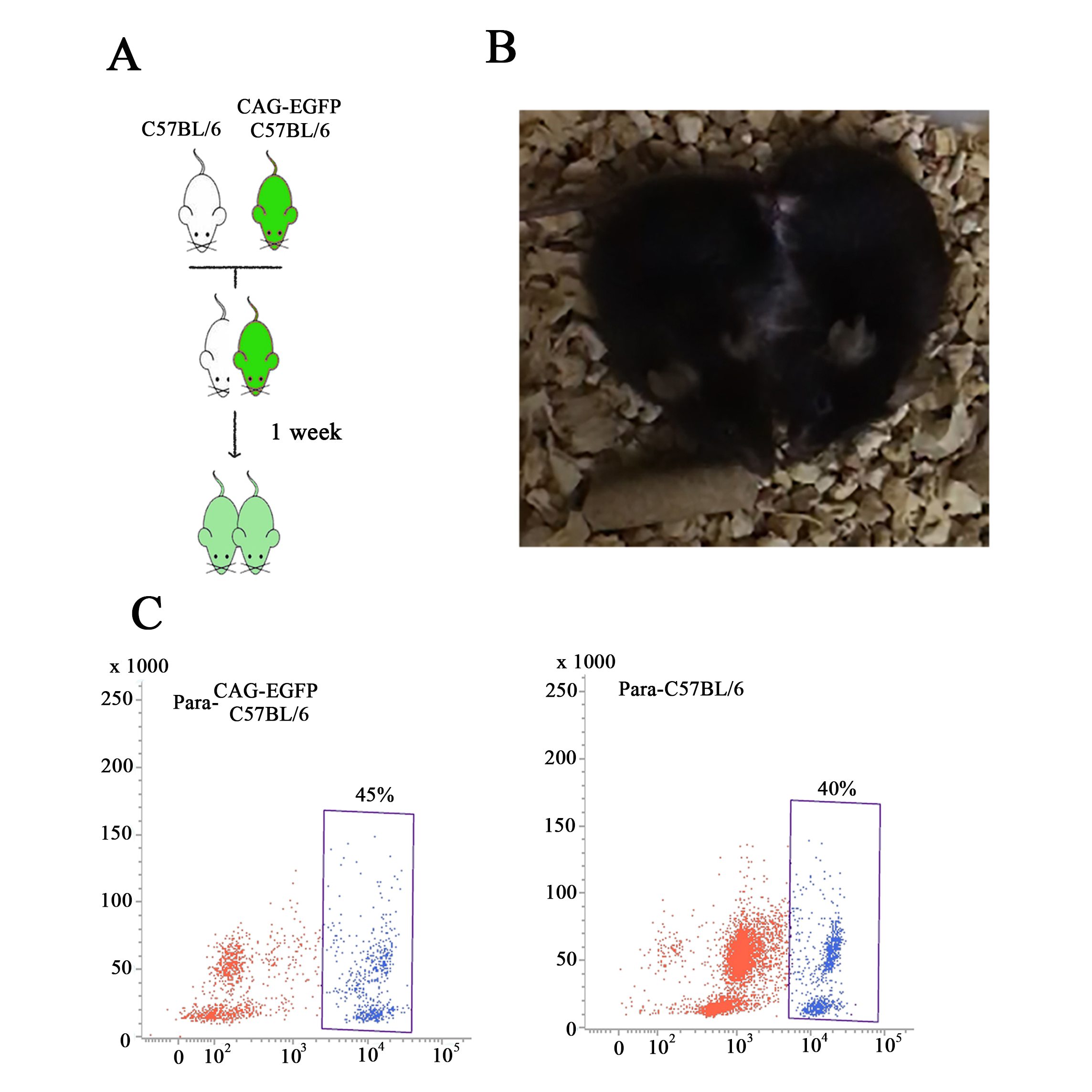

Supplement: Supplementary file 3 — Supplementary Figure 2 [file 41419_2021_4348_MOESM3_ESM.tif]

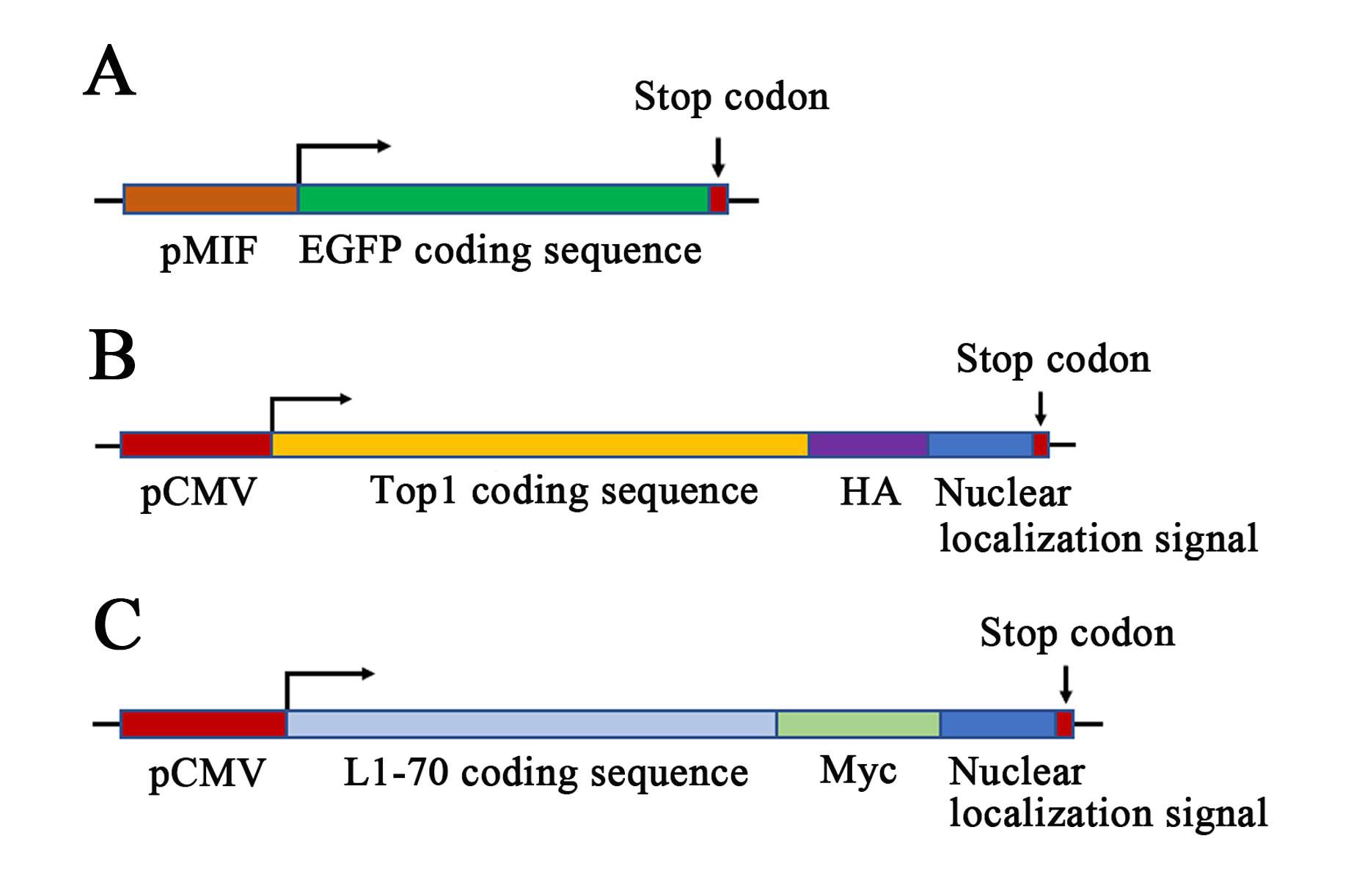

Supplement: Supplementary file 4 — Supplementary Figure 3 [file 41419_2021_4348_MOESM4_ESM.tif]
